# The role of the piriform cortex in temporal lobe epilepsy: A current literature review

**DOI:** 10.3389/fneur.2022.1042887

**Published:** 2022-11-21

**Authors:** Keanu Chee, Ashkaun Razmara, Aaron S. Geller, William B. Harris, Diego Restrepo, John A. Thompson, Daniel R. Kramer

**Affiliations:** ^1^Department of Neurosurgery, School of Medicine, University of Colorado Anschutz Medical Campus, Aurora, CO, United States; ^2^Department of Neurology, School of Medicine, University of Colorado Anschutz Medical Campus, Aurora, CO, United States; ^3^Department of Developmental and Cell Biology, School of Medicine, University of Colorado Anschutz Medical Campus, Aurora, CO, United States

**Keywords:** piriform cortex (PC), temporal lobe epilepsy, olfaction, EEG, area tempestas

## Abstract

Temporal lobe epilepsy is the most common form of focal epilepsy and can have various detrimental consequences within many neurologic domains. Recent evidence suggests that the piriform cortex may also be implicated in seizure physiology. The piriform cortex is a primary component of the olfactory network and is located at the junction of the frontal and temporal lobes, wrapping around the entorhinal sulcus. Similar to the hippocampus, it is a tri-layered allocortical structure, with connections to many adjacent regions including the orbitofrontal cortex, amygdala, peri- and entorhinal cortices, and insula. Both animal and human studies have implicated the piriform cortex as a critical node in the temporal lobe epilepsy network. It has additionally been shown that resection of greater than half of the piriform cortex may significantly increase the odds of achieving seizure freedom. Laser interstitial thermal therapy has also been shown to be an effective treatment strategy with recent evidence hinting that ablation of the piriform cortex may be important for seizure control as well. We propose that sampling piriform cortex in intracranial stereoelectroencephalography (sEEG) procedures with the use of a temporal pole or amygdalar electrode would be beneficial for further understanding the role of the piriform cortex in temporal lobe epilepsy.

## Introduction

Temporal lobe epilepsy (TLE) is the most common form of focal epilepsy and can have detrimental consequences on cognitive function including memory, language, and executive function (1). Approximately one-third of TLE patients meet the criteria for medically refractory epilepsy (i.e., fail to respond to two tolerated and appropriate anti-epileptic medications) and are considered for surgical intervention to control seizures (2, 3). Semiological, electrophysiological and imaging evidence have supported mesial temporal lobe structures, including the hippocampus, parahippocampal gyrus, and amygdala as the primary targets of surgical interventions in TLE. However, up to 40% of patients with TLE still experience postoperative seizures at 2-year follow up, suggesting that other cortical regions may have significant involvement in TLE (4). Recent evidence has suggested that the piriform cortex (PC), a primary component of the olfactory network, may also be implicated in seizure physiology. Following TLE surgery, removal of at least 50% of the PC increased the odds of achieving complete seizure freedom by a factor of 16 (95% CI, 5–47; *P* < 0.001) (5). This outcome was somewhat surprising, as the PC is rarely considered a part of the epileptogenic network. Additionally, investigation of this structure is limited as it is not traditionally sampled *via* intracranial EEG (6). Here we review the available evidence of the role the PC plays in human epilepsy. Drawing on the structure of the PC, animal studies, and limited human studies, we show that the PC is likely an important node in the TLE network, and warrants electrode placement during intracranial monitoring and surgical interventions.

## Literature search strategy

We conducted a comprehensive literature review of the available clinical and basic scientific literature from 1954 to 2022 in order to identify past and current evidence that supports the PC as being an important node in the TLE network. A browser-based search using two publicly available databases, PubMed and EMBASE, were queried for relevant articles pertaining to the PC. Notably, this work was not a systematic review, and therefore did not require adherence to the PRISMA guidelines. Each query used the search terms, “Piriform Cortex” AND “Temporal Lobe Epilepsy.” For each individual section of this review, specific search terms were added to provide context to the literature review. Additional key word search terms included “Function,” “Connectivity,” “Animal,” “Resection,” “LITT,” “MRI,” and “EEG”.

Once a broad reference list was generated for each subsection, articles were sorted based on relevance and independently evaluated for content. Additional sources were referenced from the originally selected articles that were cited for initial inclusion and further evaluated for relevant content. There were no specific article types that were excluded from this review. Only articles that were published in the English language were utilized.

## Structure and function of the piriform cortex

### Anatomical location

Defining the anatomical borders of the PC has remained a challenge. In a study conducted by Goncalves-Pereira et al., they provided extensive descriptions of the borders of the PC using Nissl-stained tissue sections. The rostral portion of the PC was described as residing in the caudolateral portion of the orbitofrontal cortex and both lateral and ventral to the lateral olfactory tract. The PC then extends toward the dorsomedial temporal lobe, around the entorhinal sulcus, and ends at the periamygdaloid region where it fuses with the cortical amygdala (7).

**Figure 1:**
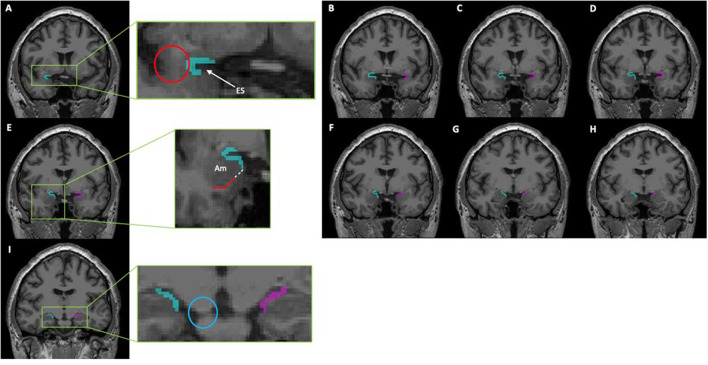
This series of coronal T1-weighted MR-images depicts a segmentation of the piriform cortex using ITK-SNAP (Version 3.8.0). From rostral to caudal, the piriform cortex can be identified as follows: **(A,B)** The most rostral portion of the piriform cortex is denoted by the presence of the limen insulae (Red Circle); here it will consist of both a frontal- and temporal-component that creates a “C” shape around the entorhinal sulcus (ES). **(C–E)** The temporal portion of the piriform cortex is progressively extended until reaching the gyrus semiannularis. If the gyrus semiannularis was not easily identified, then a parallel line (white dotted line) extending from the white matter (red solid line) under the amygdala (Am) was made toward the surface of the cortex, and the temporal piriform was extended to that point. **(F–H)** As the optic tracts move laterally and merges with the temporal cortex, the frontal component of the piriform cortex recedes correspondingly. **(I)** Only the temporal piriform cortex can be seen; the most caudal portion of the piriform cortex is seen on the the coronal slice just before the cerebellar peduncle completely merges with the pons (Blue Circle). This protocol was adapted from those previously described by Goncalves Pereira et al. (7), Galovic et al. (5), and Iqbal et al. (10).

Although they were able to delineate these anatomical margins on Nissl staining, identification of the PC on neuroimaging using these landmarks is difficult given the lack of distinct gray matter borders. Goncalves-Pereira et al. also performed volumetric MRI analyses and described a protocol in which to perform segmentation of the PC based on their histologic brain tissue evaluations. However, their protocol excluded the fPC given that it only accounts for 10–15% of the total PC volume (7). Therefore, several other subsequent studies have utilized and refined this protocol to include the fPC volume to guide surgical resection of the PC, as well as conduct additional volumetric analyses (5, 8–10). After reconciling these various protocols, we have similarly developed a segmentation protocol which includes both the fPC and tPC components. See detailed description in Figure 1.

### Cellular structure

The PC is a tri-layered allocortical structure that resides at the junction of the frontal and temporal lobe and wraps around the entorhinal sulcus (7, 11, 12). Layer I is the most superficial layer and has a high density of dendrites from cells that originate in deeper layers of the PC. Layer I is subsequently divided into a more superficial layer *Ia*, which receives primary afferent information from the olfactory bulb, and a deeper layer *Ib*, which contains recurrent innervation from PC pyramidal neurons and intracortical fibers from adjacent olfactory nuclei (13–15). Layer II is the “soma” layer that contains densely packed glutamatergic semilunar and pyramidal cells. Semilunar cells have branching dendrites that ascend into layer Ia and receive olfactory information, while pyramidal cells project apical dendrites into layer I as well as basal dendrites to layer III of the PC (16, 17). Pyramidal and semilunar cells are sparsely distributed in layer III. Layer III most prominently contains multipolar cells that are presumed to be GABAergic (16, 18). Relevant to seizure generation, in rodents there is a pronounced gradient of increasing pyramidal cell inhibition along the PC rostro-caudal axis, which may facilitate spatial activation in the anterior PC in response to various odor features (19).

Both the PC and Endopiriform Nucleus (EPN) have been implicated in TLE due to their connectivity to adjacent hyperexcitable cortical regions (20, 21). In rodents, the EPN is a cortical structure deep to the piriform cortex that contains multipolar cells that project dendrites to many areas including the PC, entorhinal cortex (ERC), insular cortex, orbitofrontal cortex, amygdala, and thalamus (21–23). The EPN is thought to be involved in the integration of olfactory and gustatory information, as well as the formation of olfactory memories and emotional learning (20). The regions of the EPN have been well delineated in rodents. However, the neuroembryological evidence for and delineated borders of EPN in humans remains unclear; whereas the EPN and claustrum appear as separate regions in rodent cortices, these structures may be a single continuous structure in humans (23). Both the PC and EPN in animal models have been shown to be hyperexcitable. The anterior EPN has a lower threshold for chemoconvulsant-induced seizures in rats (24), and application of the anticonvulsant drug, Vigabatrin, into the rat EPN, can increase the seizure threshold (25). However, further studies are needed to clarify the presence of the EPN in humans as well as precise boundaries.

### Functional divisions

The human PC is functionally divided into frontal (fPC) and temporal (tPC) subregions, which reside at the junction of the frontal and temporal lobes, rostromedial to the amygdala, and along the superior and inferior edge of the entorhinal sulcus (7, 12, 26). It has a U-shaped cross-sectional structure in the coronal view and curves around the middle cerebral artery (27). The tPC begins anteriorly at the level of the limen insulae and continues posteriorly to then overlie the amygdaloid nuclei. The PC transitions medially into the peri- or entorhinal cortex and this transition is delineated by a small depression known as the sulcus semiannularis (7, 12). The fPC extends from the fundus of the entorhinal sulcus and is bordered medially by the olfactory tubercle and lateral olfactory tract, forming a triangular region that is posterior to the orbitofrontal cortex and medial to the insular cortex (12, 27). Animal studies of macaque monkeys and rats have demonstrated that primate frontal and temporal lobe PC components correlate with the relative location of the anterior and posterior divisions of the PC in rats (12, 28). This anatomical evidence for functional differentiation between the subregions of the PC is supported by work that shows that the fPC may encode an odor's molecular features, while the tPC encodes odor quality (12). The area tempestas, located deep within the fPC (ventro-rostral aspect of the PC), is a distinct chemoconvulsant trigger zone that is uniquely susceptible to tonic-clonic seizure induction using picomole amounts of GABAergic antagonists (24). The area tempestas has histologically-demonstrated decreased GABAergic axonal input as well as decreased GAT-1 immunoreactivity, possibly explaining its hyperexcitable character (14, 20).

## Connections of the piriform cortex

### Afferent input to the PC

Olfactory processing first begins with stimulation of neurons of the olfactory epithelium, which then transmits information to mitral and tufted cells specific to their respective olfactory glomeruli (29). Mitral cells are the primary cell type that project primary olfactory afferents to the PC, while tufted cells have a less prominent role, but synapse specifically at the area tempestas; fibers from the area tempestas form the main projections to the ventrolateral orbital cortex (16, 30–32). Dendrites in layer *Ia* of the PC receive olfactory information by way of the lateral olfactory tract, which runs along the lateral surface of the fPC (16). The PC does not demonstrate any type of spatial preference or organization for incoming olfactory afferents, allowing for recognition and discrimination of a variety of different olfactory patterns and odors (13, 33). Optogenetic circuit-mapping has demonstrated that the PC also receives association fibers from other olfactory cortical areas including the anterior olfactory nucleus, frontal cortex, ERC, and the contralateral PC by way of commissural fibers (34, 35). Neuromodulatory input to the PC includes dopaminergic modulation from the ventral tegmentum, noradrenergic modulation from the locus ceruleus, and cholinergic modulation from the horizontal limb of the diagonal band (16, 27, 36).

### Efferent output to the PC

**Figure 2:**
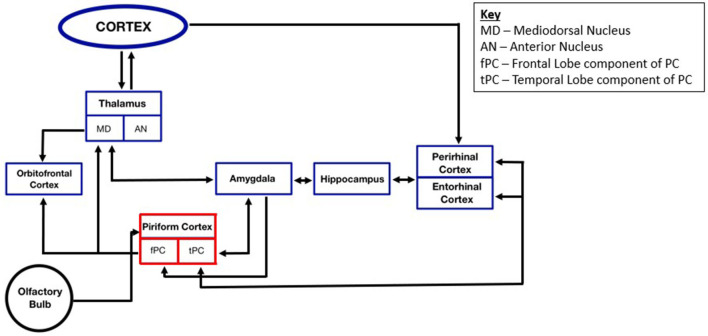
Functional overview of piriform cortex connectivity within the temporal lobe epilepsy seizure network.

With connections to the orbitofrontal cortex, amygdala, and ERC, the PC directly communicates with many of the primary locations involved in the TLE seizure network, and consequently communicates with regions targeted in traditional TLE sEEG studies (27, 37–39). The orbitofrontal cortex is the primary higher-order sensory cortex that forms reciprocal olfactory connections with the PC (40, 41). Functional neuroimaging studies, as well as frontal and temporal lobectomy studies, indicate that the orbitofrontal cortex has critical roles in odor identification, anticipation of the onset of olfactory stimuli, and integration of olfactory information, emotion, and reward value of odors and taste (27, 31, 42–44). The PC has both direct connections to the OFC, as well as indirect connections through the mediodorsal nucleus of the thalamus (28, 45). The mediodorsal nucleus of the thalamus is thought to have a role in odor discrimination and detection; however, its precise involvement in odor detection remains a topic of debate (41, 46). In humans, the amygdala's role in odor processing, elucidated through the use of positron emission tomography during odor presentation, is suggested to elicit a defensive fear response to aversive stimuli (47). Projections to the amygdala originate primarily in the tPC, and both the fPC and tPC receive reciprocal input from the basolateral amygdala nucleus, though more prominent at the tPC (39). The PC forms an important reciprocal connection with the ERC, located in the anterior parahippocampal gyrus. The ERC primarily projects to the hippocampus and serves as a relay area between the hippocampus and other sensory cortices including the perirhinal cortex and parahippocampal cortex. The ERC is involved in visuo-spatial functioning (20, 48). Finally, the insula, which is thought to include the primary gustatory cortex, has been shown to have connections with the primary olfactory cortex through tracing studies in primates and tractography in humans (49). A map depicting these various PC connections are detailed in Figure 2. A study of epilepsy patients who underwent right insular resection suggests that the insula may have a role in modulating the intensity of olfactory stimuli (50). Given the intimate network associations with these known epileptogenic structures (i.e., amygdala, hippocampus, ERC, orbitofrontal cortex, and insula), the PC constitutes a compelling locus for seizure generation and propagation.

## Role of the piriform cortex in epilepsy

### Animal studies

In animal studies, the relationship of the PC to seizure generation and propagation has been the subject of significant research for decades. In kindling models, in which repeated electrical stimulation creates after-discharges and progressively worsening severity and duration of seizures (51), the PC is the first cortical site to produce interictal discharges, regardless of where the initial seizures were generated (52). Within the PC, the anterior subregion has demonstrated faster kindling rates than the posterior subregion with no notable differences amongst the three layers of the PC's tri-layered cortex (53, 54). Long-lasting alterations in the PC include increased baseline firing rates of PC neurons with development of interictal spikes, loss of GABA-immunoreactive neurons, and paradoxical neuronal excitability induced by the normally inhibitory corticotropin releasing factor (54–57). Overall, long term and transient alterations within the PC reflect a shift to a more hyperexcitable state in the kindled rat. Kindling has also been shown to increase inhibition of non-pyramidal inhibitory cells leading to increased disinhibition of excitatory pyramidal neurons within the PC (58). Recently, kindling by optogenetic stimulation of PC (“optokindling”) was shown to elicit massive, generalized seizures in mice, which were caused by weakened feedback inhibition due to decreasing synaptic cleft GABA concentrations and slowed vesicle refilling, consistent with decreased GABA synthesis (59).

Though the precise mechanism of epileptogenesis remains unclear, ictal activity in the PC is thought to be facilitated by NMDA-mediated transmission, as application of 3,3-(2-carboxypiperazine-4-yl) propyl-1-phosphonate (a competitive NMDA antagonist) demonstrates inhibition of propagation (60). Critically, similar seizure activity can be provoked by strong olfactory input. A study on odor-induced seizure mice that express receptors to the olfactory stimulant, octanal, demonstrated tonic-clonic seizures in a subset of mice that were rapidly exposed to high concentrations of octanal. In contrast, slow increases in the concentration of octanal to a similar final concentration did not reproduce similar seizure activity (61). Therefore, it appears that a rapid increase in olfactory stimulation, with subsequent activation of many olfactory glomeruli in the olfactory bulb, can instigate seizure activity *via* the PC (60, 61).

While the exact participatory region within the PC remains to be clearly elucidated, modulatory therapy as well as lesions of the PC have shown the ability to attenuate seizure genesis in kindled animals. This implies that the PC may provide contributory regulation of limbic excitability in TLE (52, 62). The area tempestas was previously mentioned as a chemoconvulsant trigger zone deep within the fPC. The area tempestas was initially suspected to be a primary site within the PC that could be responsible for seizure genesis and propagation; however, lesions to this area alone were insufficient to decrease seizures induced by synthetic chemoconvulsants (24, 63–65). This has led to further speculation that epileptiform activity seen in the fPC may in fact originate in either the cPC or tPC. Deep cell layers within the tPC have considerably lower pre- and post-kindling after-discharge thresholds than other subregions within the PC, indicating increased susceptibility to seizure induction. The kindled tPC has also shown decreased responsiveness to antiepileptic drug treatments with a seizure threshold roughly 60–90% lower than other adjacent areas (66). Furthermore, lesions to the tPC have shown decreased spread of kindling from other epileptogenic foci. Microinjections of either GABA receptor agonists or glutamate receptor antagonists into the tPC blocked seizure propagation evoked by chemoconvulsant-induced seizures produced in the area tempestas, which further supporting the notion that the tPC may act as a crucial hub for propagating seizures (52, 67). Lesions to the cPC have also been shown to slow kindling in the amygdala (68). The cPC marks the transition zone between the anterior and posterior subregions of the PC and is partially delineated by the disappearance of the lateral olfactory tract, presence of the adjacent EPN, as well as increased prominence in layer III of the PC. Notably, there is an increase in the density of GABAergic cells in and around the cPC, which contributes to its role in regulating neighboring pyramidal cells that form excitatory synapses with other olfactory areas (62, 69). Studies have indicated that amygdala kindling in rats decreases the local density of GABAergic neurons, causing reduced inhibitory regulation of the adjacent PC subregions. Optokindling of PC weakens GABAergic inhibition, contributing to seizures (59). Effects of vigabatrin injection, an antiepileptic GABA-transaminase blocker, into all three subregions of the PC showed the greatest anticonvulsant effects in the cPC of kindled rats. Generalized seizure thresholds were markedly increased in the cPC alone, while after-discharge thresholds also showed increased levels in both the cPC and tPC (69).

Studies utilizing low frequency stimulation therapy in different subregions of the PC have shown the ability to attenuate seizure genesis and propagation in amygdala kindled rats. LFS can exert inhibitory effects during kindling acquisition and is thought to de-potentiate synaptic transmission facilitated by electrical stimulation (70). Specifically, low frequency stimulation of the cPC was found to inhibit progression of seizures through kindling stages, thereby decreasing the occurrence of seizures; however, these studies utilized monophasic low frequency stimulation pulses, and therefore this may not represent a translatable model in humans as they were not charge balanced (55, 62, 71, 72). Bayat et al. (73) showed that biphasic low frequency stimulation applied to the fPC in kainate-induced rats resulted in significant reductions in overall seizure frequency, while also completely eliminating severe seizures (Racine stage 4 and 5) in the post-stimulation period. These findings suggest that the fPC is an important target for low frequency stimulation (73).

### Human studies

TLE can present with an olfactory aura, and although this symptom does not lateralize seizure onset, it does localize it to one or more of the constituents of the olfactory sytem (74). Olfactory auras are rare among TLE patients; however, we can infer that the PC represents an important node in the genesis of these events in TLE. Olfactory hallucinations are often described as unpleasant, with the classic description being that of burning rubber. This implies activation of the PC and amygdala, both of which are similarly activated upon presentation of unpleasant odors in a non-pathological state (75).

Odor identification activates an extensive cortical network that includes the olfactory, limbic, and semantic systems. Odor discrimination primarily relies on the hippocampus, PC, and orbitofrontal cortex. Olfactory memory activates the olfactory and semantic cortices, as well as the attention systems (12). Olfaction appears to have direct connections to memory and emotion through the PC's connections to the amygdala and hippocampus. Two separate studies utilized intracranial EEG monitoring to record electrical activity in the PC during cued tasks involving either odor-related tasks or simple respiration, respectively. Odor stimulation was found to entrain theta oscillatory activity in the human PC, which is thought to facilitate the coordination of odor information between the PC and hippocampus, thereby linking the olfactory network to the limbic system (76). Nasal-respiratory flow has also been shown to be synchronized to electrical activity in the PC, amygdala, and hippocampus, suggesting that oscillatory activity in the olfactory cortex may also entrain the human respiratory cycle (77). Interestingly, passive inspiration through the nose can enhance reaction times to fearful stimuli as well as increase memory retrieval for visual object recognition (77). These data suggest that olfaction is closely tied to memory and emotion, as activity in the PC, amygdala, and hippocampus is entrained by odor stimulation and respiratory phase.

Volumetric MRI analyses have revealed olfactory cortical atrophy in patients with TLE, with a co-occurrence of volume reduction in hippocampus, amygdala, and ERC, subsequently causing impairment in odor recognition tasks (7). Greater PC atrophy was prominently noted in patients with right-sided epilepsy, though a subgroup of patients with left-sided TLE demonstrated bilateral PC atrophy. Several studies have employed combined EEG-fMRI to investigate propagation pathways of focal epileptic discharges. In a study done by Laufs et al. in 2011, 19 patients with focal epilepsy arising from all lobes demonstrated significant clusters with peak BOLD response overlying the general area of the ipsilateral PC. A subsequent study done by Flanagan et al. in 2013 similarly utilized EEG-fMRI data from 27 patients with heterogeneous epileptic foci to identify a common area of temporal lobe activation overlying the location of the ipsilateral PC. A third study performed by Fahoum et al. in 2012 compared 32 patients with TLE with 34 patients with epilepsy affecting either the frontal lobe or posterior quadrant of the cortex. The collective TLE cohort demonstrated ipsilateral activation of a network that included the insula, claustrum, temporal PC, anterior hippocampus, amygdala, mid-cingulate gyrus, and cerebellum. Importantly, neither the frontal lobe nor posterior quadrant cohorts demonstrated similar activation of the PC on EEG-fMRI imaging (12, 78–81). Collectively, EEG-fMRI data from these studies localizes abnormal activity in patients with TLE to an area that includes ipsilateral PC.

With the development of new surgical techniques and technologies, surgery in epilepsy has greatly evolved in the past three decades. The advancements in neuroimaging, as well as development of various tools, particularly in the field of neuro-navigation, have significantly aided neurosurgeons' abilities to surgically manage patients with TLE (82). Information derived from electrophysiologic data, diffusion tensor imaging, and MRI, among other modalities, has improved the visualization of various target structures which have yielded promising outcomes in several studies. Galovic et al. performed volumetric MRI analysis on 107 patients with unilateral TLE following standard anterior temporal lobe resection and concluded that removing at least 50% of the PC increased the odds of achieving complete seizure freedom by a factor of 16. In contrast, removal of adjacent cortical areas including the hippocampus, amygdala, and entorhinal cortex had no significant correlation with achieving seizure freedom (5). Borger et al. employed a similar study design and evaluated patients who underwent transsylvian selective amygdalo-hippocampectomy that included the PC. By utilizing the International League Against Epilepsy classification scale, patients were stratified as having either favorable (ILAE class 1) or unfavorable (ILAE class 2–6) post-surgical seizure outcomes. Patients who had favorable seizure outcomes had a greater proportion of the PC resected (51%) compared to patients who had unfavorable outcomes (13%, *p* < 0.0001). Again, the degree of the hippocampal and amygdalar resections did not differ between the patient groups (8). Tyrand et al. found that patients with treatment resistant TLE had significant decreases in interictal epileptic discharge rates (IEDR) following anterior temporal lobe resection, specifically with resection of the superior temporal gyrus. Additionally, 100% of patients who underwent concomitant mesial resection remained seizure free at 12-month follow up (83); such findings strongly advocate for inclusion of both anterior and mesial temporal lobe structures in the parameters for surgical management of TLE patients.

Laser interstitial thermal therapy (LITT) has also been shown to provide an effective and minimally invasive treatment modality for patients with TLE. Wu et al. analyzed the efficacy of LITT in 234 patients with TLE and reported that 58-percent of patients were completely seizure free, and 76-percent of patients were either completely seizure free or almost seizure free at the time of last follow up. Contrary to both studies by Galovic and Borger, this study emphasized that the margins of laser-ablation should include the amygdala, hippocampal head, parahippocampal gyrus, and rhinal cortices to maximal seizure freedom. However, while it does appear that some PC volume was included in the distribution of ablation, no clear inclusion parameters for the PC were specified (84). Kerezoudis et al. conducted a meta-analysis that included a total of 551-patients who received LITT ablation of the hippocampus and amygdala for treatment of TLE and mesial temporal lobe sclerosis. In this cohort, the average ablation margins included 67.5% of the hippocampus and 58.7% of the amygdala. Overall seizure freedom achieved in patients following ablation was 58% (95% CI, 54–62%), though their results did not demonstrate statistical significance with regards to total ablation volume, hippocampal ablation, or amgydalar ablation (85). Hwang et al. conducted a pre- and post-ablation volumetric analysis on patients with mTLE who underwent magnetic resonance guided LITT of the hippocampus, amygdala, and piriform cortex to determine the relationship between ablation volume and seizure outcomes. Following multivariate logistic regression analysis, they determined that in patients with mTLE, percent PC ablation volume was a significant predictor of seizure freedom at both 6 months (95% CI = 1.012–1.193; *p* = 0.019) and 1 year (95% CI = 1.003–1.178; *p* = 0.041) (86). Although these studies suggest that the PC may be an important target to include in ablation volumetric parameters, no studies have explicitly defined such margins.

## Future directions

### Stereo-encephalography

Given the demonstrated effects of PC resection on rates of postoperative seizure freedom, further studies on the role of the PC in seizure generation and propagation are needed. Since tPC resection specifically has been shown to be associated with a higher seizure freedom rate (5), sampling the tPC with an intracranial EEG electrode is likely warranted; this is already being done for olfactory research (87). The corresponding sEEG data may provide more clarity regarding the PC's role in epileptogenesis, as well as more rationale for incorporating the PC as a standard site for intracranial EEG monitoring. Additionally, this data can be further utilized to guide surgical resection or laser ablation of the PC in patients with medically refractory TLE.

**Figure 3:**
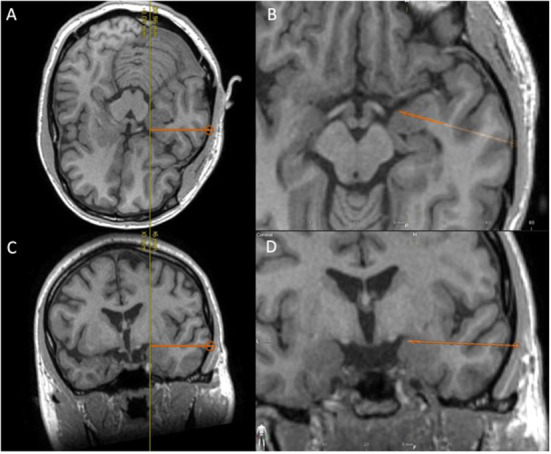
Using T1-weighted MRI, we demonstrate the proposed placement of a temporal electrode that would end in the temporal piriform cortex; **(A)** Peri-axial in-line view, **(B)** enlarged peri-axial view, **(C)** Peri-coronal in-line view, and **(D)** enlarged peri-coronal view.

We propose that the trajectory of an existing temporal pole or amygdalar electrode can be slightly altered and positioned in such a way as to terminate in the tPC (Figure 3). Despite the complication rates associated with sEEG implantation being low, this trajectory does have a theoretical risk for causing hemorrhage, or neurologic deficits (88, 89). Specifically, with hemorrhagic complications representing the most common complication associated with sEEG implantation (89), this trajectory does pose some risk of having the electrode end up in the cistern where it can potentially compromise cisternal vessels or even damage cranial nerves. However, our suggestion is to follow a slightly elevated trajectory that would ensure entrance into the tPC near the frontotemporal junction, where the risks of causing significant damage to the cisternal vessels or cranial nerves are minimal. Whether an additional electrode targeting the fPC is necessary remains to be determined.

### Laser ablation

Roughly 50–55% of patients undergoing laser ablation for treatment of TLE remain seizure free (90, 91). The preferred laser fiber trajectory is aimed more superiorly in the uncus in order to penetrate the central portion of the hippocampus and continue through the amygdala to reach the medial temporal pole. The primary targets for laser ablation include the hippocampus, subiculum, amygdala, and uncus (91). The volume of ablation may very well include the same portion of the PC as is noted in surgical resection patients; however, the PC is not explicitly targeted in such procedures. Further studies that specifically examine the volumetric ablation parameters of the PC should be done in laser-ablation cohorts to determine whether seizure freedom rates in patients who undergo surgical resection can be replicated following LITT.

### Lack of seizure freedom after surgery

Similarly, reviewing cases of poor seizure freedom outcomes in resection or ablation cases for remaining PC tissue may be worthwhile. As the PC marks the location where the amygdala transitions into the deep frontal lobe, many mesial temporal resections are purposefully not taken too superiorly within the uncus for fear of violating the basal ganglia or the anterior perforating substance. More extensive resection, laser ablation, or even focused ultrasound that targets that remaining tissue may be a reasonable option for such patients.

### Studies of PC activity in humans

Further research is required to better understand the role of the PC in seizures. Studies utilizing functional MRI, magnetoencephalography, sEEG, and even electrodes capable of single-unit resolution are warranted to gain a deeper insight as to specific mechanisms of seizure transmission within the PC. Such knowledge can potentially be leveraged for targeted treatment modalities, both pharmacologic and surgical.

## Discussion

This comprehensive literature review summarizes the structure, function, and involvement of the PC within the limbic network, which ultimately make the PC a region of high susceptibility for seizures (12, 20, 27).

Early work by Piredda and Gale identified a specific locus within the animal fPC known as the *area tempestas*, an area from which seizures could be elicited following just picomole amounts of chemoconvulsant administration (24). Additional animal studies showed that the PC is the first cortical site to develop interictal discharges following kindling, and repeated stimulation can result in neurons transitioning to a hyperexcitable state in response to normally inhibitory neurotransmitters (52, 54–57). This work highlights the ability of the PC to serve as both a sight of seizure generation, as well as a site of seizure propagation throughout the limbic network.

Various studies utilizing either antiepileptic pharmacotherapy or lesion induction in the animal PC have also shown the ability to reduce seizure propagation (52, 67, 68). Low frequency stimulation has demonstrated good efficacy in inhibiting seizure progression through kindling stages in mice; however, whether these results are translatable to human research depends on whether investigators utilize monophasic or biphasic low frequency stimulation (55, 62, 70–73). Given that both chemical and electrical stimulation of the PC can result in seizures in animals, while antiepileptic pharmacotherapy, lesion-inducing therapy, and low frequency stimulation are all capable of diminishing seizure generation or propagation in animals, there is a clear role for the PC in TLE.

In the context of human TLE, the PC has been steadily gaining more attention as a critical site of epileptogenesis and seizure propagation. The olfactory hallucinations that patients often describe before the onset of seizures were some of the first implications that the olfactory cortex, and specifically the PC, were involved in TLE. Anatomical and volumetric studies were able to delineate the borders of the PC using histologic analyses (7). Additionally, various PC segmentation protocols were then developed and refined in order to reliably identify the PC using neuroimaging and perform volumetric analyses to determine PC volume reduction in the setting of TLE (5, 7, 9, 10). These protocols may have future utility in guiding depth electrode placement for sEEG studies, as well as facilitating surgical resection for definitive treatment of TLE.

Several studies that utilized EEG-fMRI to study brain activity in patients with TLE consistently observed abnormal activity in an area corresponding with the PC (12, 78–81). Additionally, various studies have reported excellent rates of seizure freedom following surgical resection of the PC (5, 8, 83). Laser interstitial thermal therapy is an alternative therapeutic option that has demonstrated good efficacy in the treatment of patients with medically refractory TLE (84–86); however, future studies should aim to define specific inclusion parameters for the PC within the distribution of LITT ablation.

Collectively, both animal and human studies have shown that the PC is important in TLE. The studies reviewed here support the notion that the PC is a common node in epilepsy and targeting the PC in temporal procedures provides a higher rate of seizure freedom. As we accrue more scientific data pertaining to the unique role and function of the PC in epilepsy, this may ultimately provide new insights into improving the treatment and management of TLE.

## Author contributions

Primary development of the manuscript with substantial contributions to the conception or design of the work: KC and DK. Drafting the work and/or revising it critically for important intellectual content and agreement to be accountable for all aspects of this work: KC, AR, WH, AG, DR, JT, and DK. Final approval of the version to be published: KC, JT, and DK. All authors contributed to the article and approved the submitted version.

## Conflict of interest

The authors declare that the research was conducted in the absence of any commercial or financial relationships that could be construed as a potential conflict of interest.

## Publisher's note

All claims expressed in this article are solely those of the authors and do not necessarily represent those of their affiliated organizations, or those of the publisher, the editors and the reviewers. Any product that may be evaluated in this article, or claim that may be made by its manufacturer, is not guaranteed or endorsed by the publisher.
