# Corrigendum: The role of the piriform cortex in temporal lobe epilepsy: A current literature review

**DOI:** 10.3389/fneur.2023.1148230

**Published:** 2023-02-23

**Authors:** Keanu Chee, Ashkaun Razmara, Aaron S. Geller, William B. Harris, Diego Restrepo, John A. Thompson, Daniel R. Kramer

**Affiliations:** ^1^Department of Neurosurgery, School of Medicine, University of Colorado Anschutz Medical Campus, Aurora, CO, United States; ^2^Department of Neurology, School of Medicine, University of Colorado Anschutz Medical Campus, Aurora, CO, United States; ^3^Department of Developmental and Cell Biology, School of Medicine, University of Colorado Anschutz Medical Campus, Aurora, CO, United States

**Keywords:** piriform cortex (PC), temporal lobe epilepsy, olfaction, EEG, area tempestas

In the published article, there was an error in Figure 2 as published. An older version of the figure, with differences in formatting, appeared in the published article. The figure caption has also been updated from “Functional overview of piriform cortex connectivity within the TLE seizure network” to “Functional overview of piriform cortex connectivity within the temporal lobe epilepsy seizure network.” The corrected Figure 2 appears below.

**Figure 2:**
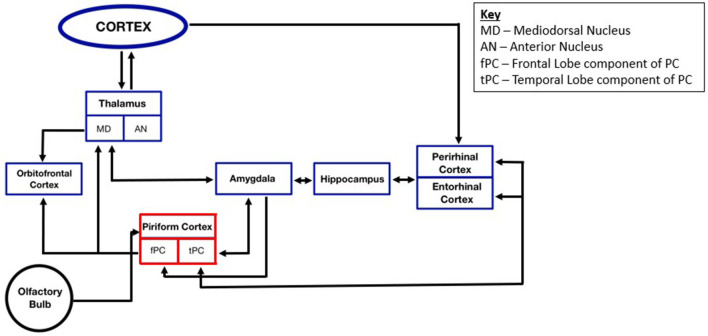
Functional overview of piriform cortex connectivity within the temporal lobe epilepsy seizure network.

The authors apologize for this error and state that this does not change the scientific conclusions of the article in any way. The original article has been updated.

